# Extension of mandatory health insurance to informal sector workers in Togo

**DOI:** 10.1186/s13561-018-0208-4

**Published:** 2018-09-17

**Authors:** Dosse Mawussi Djahini-Afawoubo, Esso-Hanam Atake

**Affiliations:** Department of Economic Sciences, University of Lomé (Togo), Lome, West africa Togo

**Keywords:** Informal sector worker, Health insurance, Contingent valuation, Willingness-to-pay, Togo

## Abstract

**Background:**

About 90.4% of Togolese workers operate in the informal sector and account for between 20 and 30% of Togo’s Gross Domestic Product. Despite their importance in the Togolese economy, informal sector workers (ISW) do not have a health insurance scheme. This paper aims to estimate the willingness-to-pay (WTP) of ISW in order to have access to Mandatory Health Insurance (MHI), and to analyze the main determinants of WTP.

**Methods:**

This study used data from the Community-Based Monitoring System (CBMS) project implemented in 2015 by the Partnership for Economic Policy (PEP). It focusses on 4,296 ISW (2,374 in urban areas and 1,922 in rural areas, respectively). The contingent valuation method was used to determine the WTP for the MHI while the Tobit model is used to analyze its determinants.

**Results and discussion:**

Findings indicate that about 92% of ISW agreed to have access to MHI, like for formal sector workers. Overall, ISW are willing to pay 2,569 FCFA (USD 4.7) per month. ISW in the poorest quintiles are willing to allocate a higher proportion of their income (15%) to the premium than the richest quintiles (2.5%). Generally, women are more interested in MHI than men, although men are willing to pay higher premiums (3,168.9 FCFA or USD 5.8) than women (2,077 FCFA or USD 3.8). Women’s lower WTP can be explained by their low levels of education and income, and a lack of employment opportunities compared to men. The gender of the head of the household, the size of the household and the education and income levels are the main determinants of WTP.

**Conclusion:**

We conclude that it is possible to extend MHI to ISW as long as their premiums are subsidized. The annual subsidy is estimated at 4.1% of the state current general budget or 96% of the health sector budget. In setting the premium, policy makers should take into account the MHI benefits package, subsidies from the government, and information about the WTP. It is important to emphasize that resource mobilization and management, as well as health services delivery, would be effective only in a context of improved governance.

## Background

Access to health care can be extremely expensive and few people can afford to pay their own medical expenses [1, 2]. Governments should put in place policies that enable people to access health care services without incurring catastrophic health expenditures. Universal health insurance may therefore be a fundamental tool for improving people’s welfare [1]. Access to universal health insurance could impact households in a number of ways. Firstly, it would lead to better health by protecting households from health shocks that reduce their capacity to generate income [3]. Secondly, access to health insurance is supposed to reduce out-of-pocket payments [4, 5]. In other words, households that are not insured have to allocate a large part of their budget to solving health problems which reduces the resources available for other goods [6, 7]. Finally, health insurance is essential in supporting the high costs of childbirth [8]. Access to health insurance should, therefore, contribute greatly to the individual and collective well-being.

Unfortunately, in developing countries, experiences have shown that it is difficult to group risks by including vulnerable populations such as Informal Sector Workers (ISW) into existing health insurance schemes [9]. The informal sector is characterised by low and irregular incomes which make it difficult to pay for health care in advance [10]. The existing prepayment systems, including government financing in least developed countries, exclude informal sector workers either because of inadequate resources or because they are too weak to afford the insurance premiums [10, 11]. This problem is particularly crucial in Togo, a Sub-Saharan African country ranked among the least developed countries.

The analysis of the determinants of the ISW’s willingness to pay for Mandatory Health Insurance (MHI) is a major concern in the case of Togo for at least two reasons. First, a significant proportion of the Togolese workforce (90.4%) works in the informal sector [12]. Secondly, the utilization of health care services is very limited in Togo because of the high out-of-pocket payments [13]. Despite their importance in the Togolese economy, ISW do not have health insurance. In order for a greater number of workers to benefit from health insurance, a MHI scheme was instituted by Law n° 2011–003 of 18 February 2011. The scheme’s main objective is to provide better financial access to quality healthcare for its beneficiaries. Currently, ISW are excluded from this health insurance scheme. As a result, it covers only 4% of the Togolese population [14]. In order to extend the MHI to ISW, it is necessary to determine their WTP for a health insurance premium and to identify the determinants of their WTP. Indeed, if the premium amount is higher than what ISW are willing to pay, they would be disadvantaged and a real problem of social justice would arise. The identification of the determinants of informal sector workers’ willingness-to-pay for health insurance could help policymakers in setting the premium. The objective of this paper is to examine the determinants of ISWs’ willingness to pay for MHI as is the case for formal sector workers in Togo. The paper aims specifically to estimate the ISWs’ willingness to pay for MHI, and to analyze the main determinants of the WTP.

Several studies have analyzed the determinants of WTP for health insurance [12, 15–18]. Donfouet et al. [16] investigated the determinants of WTP for a community-based prepayment healthcare scheme in rural Cameroon using a contingent valuation method. They found that age, religion, knowledge of community-based health insurance, awareness of usual practice in rural areas, involvement in association and disposable income are key determinants of WTP. Dong et al. [17] and Onwujekwe et al. [18] found that WTP is influenced by household characteristics, such as location, household size and age composition. However, very few studies have focused on the specific case of ISW [18, 19]. Bärnighausen et al. [15] analyzed ISWs’ willingness to pay for a health insurance scheme in China. They found that income and past expenditures on healthcare had a positive effect on WTP. They also found that being male, a migrant, or without permanent employment significantly decreased WTP. As for Atake and Agbodji [12], they analyzed the ISW’s willingness to pay for social protection in Togo. Their results reveal that 90.9% of Togolese ISW are willing to join social protection services. They also found that income and education were the main determinants of WTP for social protection.

The present paper differs from Atake and Agbodji [12] for at least two reasons. First, Atake and Agbodji [12] analyzed the WTP for a social protection scheme. Unfortunately, the Togolese social protection scheme includes only benefits available to households with children, pensions, workplace accidents and occupational diseases. Consequently, health insurance has been neglected. The second difference is methodological. Atake and Agbodji [12] used a logit model to analyze the determinants of ISWs’ willingness to pay for social protection in Togo. However, in their analysis, it was important to consider the censored nature of the dependent variable since some individuals may not be willing to pay. Under these circumstances, a Tobit model would be more appropriate.

### Overview of the social protection system and the health insurance scheme in Togo

The social protection scheme in Togo is offered by the National Social Security Fund (CNSS) and the National Pensions Fund (CRT). Private sector workers depend on the CNSS while civil servants depend on the CRT. The benefits cover only three domains namely: households with children, pensions (invalidity, old age, death and the survivors), workplace accidents and occupational diseases. The law on the social security code in Togo was adopted by the National Assembly in 2011 (Law n° 2011–006 enacted on 21 February 2011). This law theoretically extends social protection to ISW. However, it is yet to be applied by the CNSS. As a result, ISW are still deprived of social security protection. The implementation of the MHI offered by the National Health Insurance Institute (INAM) in 2012 has not been favorable to ISW. In 2012, INAM covered approximately 300,000 people for a population of more than 6,000,000 individuals. Its mission is to provide coverage of risks related to illness, non-professional accidents and motherhood to its main beneficiaries who are workers of public administrations, and their legal beneficiaries: civil servants, magistrates, military and paramilitary forces, contract staff working with parastatals and local government officials. It also includes members of the institutions of the Republic during their term in office. In order to meet the requirements of universal health insurance, INAM plans to extend its services in the coming years to ISW and vulnerable population. In this regard, it is important to set up a healthcare financing mechanism that will allow ISW to be involved in the health insurance program.

## Methods

### Data

#### Study setting

This study uses data from the Community-Based Monitoring System (CBMS) project implemented in 2015 by the Partnership for Economic Policy (PEP), in partnership with the Department for International Development (DFID) of the United Kingdom (UK Aid) and the International Development Research Center (IDRC) of Canada. The main objective of the project was to set up a system to locally monitor the various dimensions of poverty in the informal sector, in an attempt to explore feasibility of a social protection mechanism that would provide sufficient financial protection for workers in the informal sector. Data were collected in a district (Tokoin-Wuiti) of Lomé, the capital of Togo, and in two rural cantons, namely Gblainvié and Dalavé not far from Tokoin-Wuiti. These two rural communities are characterised by poor housing conditions, poor access to safe drinking water, poor hygiene and sanitation conditions, high unemployment and informal employment rates, low levels of education, and high prevalence of disease. Tokoin-Wuiti is an urban area located near industrial and commercial areas and comprises high and middle-income households with acceptable conditions of hygiene, sanitation and housing. This area is also characterised by better access to public infrastructure (health, education, market, etc.) and a high rate of informal employment.

### Data collection

The CBMS data is all the more important for policy development as it left no one behind. In other words, data were collected from all households in the target localities. This data collection strategy makes it possible to generalize the results at the community and national levels. The three areas concerned included a total of 7846 households. In total, the data were collected from 7436 households, including 4296 ISW (2374 in urban areas and 1922 in rural areas, respectively). Uninhabited houses and refusals to answer questionnaires accounted for about 5.22% of the target households. The data were collected by interviewers who were originally from these areas, in order to facilitate questionnaire administration in the local language or mother tongue in case the respondent was illiterate. The final questionnaires were first prepared in French and then translated into the local languages during the training of enumerators. Trained enumerators conducted in-person, face-to-face interviews. They went into every household and interviewed only the heads of household or their spouses.

Three survey questionnaires were submitted to the respondents: the household profile questionnaire (HPQ), the rider questionnaire (individual questionnaire) and the community profile questionnaire (CPQ). The HPQ included information on household/member characteristics, education, health and nutrition, housing and tenure, water sources and sanitation, etc. The RQ was designed to collect additional information on social protection, and included an assessment of the ISWs’ willingness to pay for access to social protection services such as health insurance, old-age social security, workmen’s compensation, maternity benefits, etc. As soon as the respondents were identified as informal sector workers, they were asked to answer the RQ. Finally, the CPQ was responded by chiefs/representatives of districts/areas and by resource persons in health, education and other sectors. The CPQ contains information at the community level that was used to complement the information from the HPQ, such as physical and demographic characteristics of the village/community, demographic reference, service institutions and infrastructure, programs, projects and activities, etc.

### Main variables

The RQ was used to collect data on the income of household members during the last four weeks preceding the survey. For workers with a daily or weekly income, we adjusted the income to its monthly value using an appropriate multiplier (26 or 04 respectively). Income data was collected in WAEMU francs (CFA francs). The average exchange rate during the survey period, July 2015 and September 2015, was 550 CFA francs per US dollar. With regard to data on social protection and willingness to pay, this study used the parametric approach of the referendum format. According to Arrow et al. [20], the referendum “refers to a choice mechanism that asks each respondent how he would vote if faced with a particular program and the prospect of paying for the program through some means such as higher taxes”. First, the interviewers explained at length the advantages of each type of social protection service, using the national language and/or mother tongue. Information was then collected on the value they attributed to each social protection service, their availability to access it and the value of their willingness to pay. If the respondent was interested in the social protection services offered and answered “yes” to the first price proposed, the interviewer increased the offer slightly until the respondent answered “no”. If the initial answer was “no” to the first price proposed, the interviewer gradually reduced the amount until the respondent answered “yes”.

### Empirical strategy

In order to extend the mandatory health insurance scheme to ISW, in a context characterized by an insufficient budget and where barely 4% of the population is covered, it is important to hear from the household heads working in the informal sector about their willingness or unwillingness to participate in the mandatory health insurance scheme and the maximum amount they are willing to pay (WTP).

Generally, the WTP of a household head depends on his own characteristics, the characteristics of his/her household and the availability of health services. In the light of previous studies [9, 15, 21], WTP is expected to increase with age [22], income, and education [23]. Other variables such as household composition (measured by the number of adults aged over 60 and the number of children aged under five), marital status of the household head, sex of the household head, as well as distance to the nearest health facility are also included. This last variable is introduced to take into account the availability of health facilities. Thus, WTP is expected to decrease proportionally to the increase in distance to the closest health facility. The general model is as follows:

1 $$ WTP=f\left( AGE, GENDER, EDU, MAT, COMP, INCOME, RESIDENCE, DIST\right) $$

with WTP, the willingness to pay; *AGE*, the age of the household head; *GENDER*, the sex of the household head; *EDU,* the educational level of the household head; *COMP*, the household composition; *MAT*, the marital status of the household head; *INCOME*, the income level; *DIST*, the distance to the nearest health facility, *RESIDENCE*, the location of residence.

Because some individuals may not be willing to pay for pre-financing healthcare, our dependent variable (WTP for MHI) is a censored variable. Under these circumstances, the use of the ordinary least squares method is inappropriate because it leads to biased and inconsistent estimates [24]. A limited dependent variable model is therefore more appropriate. For this reason, the Tobit model [25] is chosen. The econometric model of willingness-to-pay for the MHI is then:

2 $$ {L}_h={X^{\hbox{'}}}_h\beta +{\xi}_h $$

where *L*_*h*_ is a latent variable close to the maximum premium that a household head *h* is willing to pay. We assume that *L*_*h*_ linearly depends on *X*_*h*_ -the household head’s characteristics and the characteristics of his/her household as well as the availability of health facilities (distance to the nearest health facility as described above) - via a parameters vector *β*;*ξ*_*h*_ is a normally distributed error term (with average equal to zero and a constant variance σ^2^) to capture random influences on this relationship.

Let us suppose that *Y*_*h*_ is the total premium that an individual *h* is willing to pay. *Y*_*h*_ (the observed variable) is defined to be equal to the latent variable whenever the latent variable is above zero and zero otherwise.

3 $$ {Y}_h=\left\{\begin{array}{l}{X^{\hbox{'}}}_h\beta +{\xi}_h\kern1em if\kern1em {L}_h\succ 0\\ {}0\kern4em if\kern1em {L}_h\le 0\end{array}\right. $$

The Tobit model makes it possible to value the parameters *β* and σ^2^ from the observations of *Y*_h_ and *X*_*h*_. Since the total amount of WTP *Y*_*h*_ can be either positive or zero, the likelihood function can be expressed as follows:

4 $$ L\left(\beta, {\sigma}^2/Y,X\right)=\prod \limits_{Y_h=0}\left[1-\Phi \left(\frac{{X^{\hbox{'}}}_h\beta }{\sigma}\right)\right]\prod \limits_{Y_h>0}\left[\frac{\varphi \left[\left({Y}_h-{X^{\hbox{'}}}_h\beta \right)/\sigma \right]}{\sigma}\right] $$

Where **Ф** and **φ** represent the distribution function and the density function respectively.

## Results

### Descriptive statistics

Table 1 shows some socio-demographic characteristics of Togolese ISW households. The data indicates that overall, ISW aged 15 to 35 represent 48.4%. The following category is aged 35 to 60 (45.1%). People over 60 years of age represent only 6.4%. Most households had between 2 and 4 persons (56.9%). Nearly 70% of ISW are married, compared to 16% who are single. We also note that there are more widows than widowers. This result can be explained by two phenomena, namely higher male mortality and the age gap at first marriage. According to data from Togo’s National Institute of Statistics and Economic and Demographic Studies (INSEED) [26], women’s life expectancy at birth is 64.2 years compared to 56.4 years for men [27]. Similarly, women marry earlier than men; the age at first marriage for men is 25.0 years compared to 19.7 years for women [27].

**Table 1:** Socio-demographic characteristics (%)

Characteristics	Urban			Rural			Total		
	Men	Women	All	Men	Women	All	Men	Women	All
Characteristics of household head									
*Age*									
15–35	56.8	52.3	54.0	41.4	41.4	41.4	49.1	47.9	48.4
35–60	40.4	43.6	42.4	48.9	48.1	48.5	44.7	45.4	45.1
More than 60 years	2.7	4.0	3.6	9.6	10.4	10.0	6.2	6.6	6.4
*Educational level*									
None	10.7	31.0	23.5	32.9	67.8	51.6	21.9	45.8	36.0
Primary	18.4	29.0	25.1	34.3	22.6	28.0	26.4	26.4	26.4
Secondary	59.3	38.1	45.9	32.3	9.5	20.1	45.8	26.6	34.5
University	11.5	1.9	5.4	0.4	0.1	0.3	6.0	1.2	3.1
*Marital status*									
Single	33.9	16.8	23.1	9.3	5.1	7.1	21.6	12.1	16.0
Married	60.7	66.2	64.2	84.5	70.3	76.9	72.6	67.8	69.8
Divorced or separated	3.9	7.5	6.2	3.6	6.3	5.1	3.7	7.0	5.7
Wido*w*/widower	1.5	9.5	6.5	2.6	18.2	11.0	2.0	13.0	8.5
Household size									
Single	53.4	34.4	41.4	23.8	21.0	22.3	38.6	29.0	33.0
2 to 4	38.9	56.5	50.1	64.0	66.8	65.5	51.5	60.6	56.9
4 to 6	6.3	7.9	7.3	10.6	9.8	10.2	8.5	8.6	8.6
more than 6	1.2	1.2	1.2	1.6	2.5	2.1	1.4	1.7	1.6

Concerning education, the results show that most Togolese ISW (36%) have no educational level. 26.4% have primary school education, 34.5% secondary school education and only 3.1% have university education. Furthermore, Table 1 shows that 45.8% of women have no educational level compared to 21.9% of men. There are some gender-based disparities. The results show also that as the level of education rises, the gap between men and women increases. These disparities can be explained by the inequalities of retention between girls and boys in the school system. The low retention of girls can be explained by early pregnancies that increase their dropout rate compared to boys. In addition, some customary practices predispose girls to domestic work, which can increase their rate of failure and dropout compared to boys. The same customary practices explain the fact that girls most often drop out of school to be economically active in order to help the household when the need arises.

### Willingness to pay

Table 2 presents descriptive statistics of income and WTP. It shows that on average an ISW earns a monthly income of 59,726 FCFA (USD 108.6), or 1.71 times the guaranteed minimum wage (SMIG). Men earn a higher income (62,490.9 FCFA or USD 113.6) than women (57,076 FCFA or USD 103.8). Moreover, ISW in urban areas earn on average a higher income (72,734.6 FCFA, or USD 132.2) than those in rural areas (42,263.3 FCA or USD 76.8). However, overall, Togolese ISW are willing to pay 2569 FCFA (or USD 4.7) per month on average to be involved in MHI. This amount represents about 4.3% of their average monthly income. On average, men are willing to pay a higher premium (3168.9 FCFA or USD 5.8, which represents 5.1% of their average monthly income) than women (2077 FCFA or USD 3.8, which represents 3.6% of their average income). Similarly, ISW in urban areas are willing to pay on average a higher premium (2860 FCFA, or USD 5.2, which represents 3.9% of their income) than those living in rural areas (2116.4 FCFA or USD 3.8, which represents 5% of their income).

**Table 2:** Descriptive statistics of income and WTP by quintile

	Income (in US Dollar)			WTP (in US Dollar)			Average WTP in percentage of average income
	Mean	Max	Min	Mean	Max	Min	
All households	108.6 (161.6)	3930.9	0.0	4.7 (7.7)	72.7	0.0	4.3
Quintile 1	22.8 (10.1)	36.4	0.0	3.4 (7.4)	72.7	0.0	15.0
Quintile 2	54.2 (6.4)	63.6	37.9	3.6 (5.6)	72.7	0.0	6.7
Quintile 3	81.9 (7.9)	90.9	65.5	4.3 (5.1)	29.1	0.0	5.2
Quintile 4	113.3 (10.3)	136.4	91.6	4.4 (5.1)	29.1	0.0	3.8
Quintile 5	283.8 (306.5)	3930.9	140.0	7.2 (11.9)	72.7	0.0	2.5
Men							
All men	113.6 (172.5)	3930.9	0.0	5.8 (9.7)	72.7	0.0	5.1
Quintile 1	23.6 (9.9)	36.4	0.0	5.0 (10.4)	72.7	0.0	21.3
Quintile 2	54.5 (6.5)	63.6	37.9	4.5 (5.3)	29.1	0.0	8.2
Quintile 3	82.7 (7.8)	90.9	65.5	5.0 (5.9)	29.1	0.0	6.0
Quintile 4	113.4 (10.5)	136.4	91.6	4.8 (5.4)	29.1	0.0	4.3
Quintile 5	273.2 (313)	3930.9	140.0	8.4 (14.9)	72.7	0.0	3.1
Women							
All women	103.8 (150.3)	2236.4	0.0	3.8 (5.4)	72.7	0.0	3.7
Quintile 1	22.2(10.1)	36.4	0.0	2.2 (2.8)	15.3	0.0	9.7
Quintile 2	53.9 (6.3)	63.6	38.2	3.1 (5.7)	72.7	0.0	5.7
Quintile 3	81.0 (7.9)	90.9	65.5	3.8 (4.3)	15.3	0.0	4.6
Quintile 4	113.2 (10.1)	136.4	92.7	3.9 (4.9)	29.1	0.0	3.5
Quintile 5	297.7 (297.7)	2236.4	141.8	5.9 (7.0)	29.1	0.0	2.0
Urban							
All urban	132.2 (181.7)	3930.9	0.0	5.2 (7.9)	72.7	0.0	3.9
Quintile 1	25.2 (11.3)	36.4	0.0	2.3 (3.0)	15.3	0.0	8.9
Quintile 2	55.1 (6.0)	63.6	37.9	3.7 (4.8)	29.1	0.0	6.8
Quintile 3	82.5 (7.6)	90.9	65.5	4.6 (5.5)	29.1	0.0	5.6
Quintile 4	113.8 (10.2)	136.4	92.7	4.8 (5.3)	29.1	0.0	4.2
Quintile 5	276.6 (304.7)	3930.9	140.0	7.7 (11.9)	72.7	0.0	2.8
Rural							
All rural	76.8 (122.9)	2181.8	0.0	3.8 (7.4)	72.7	0.0	5.0
Quintile 1	22.2 (9.6)	36.4	0.0	3.8 (8.3)	72.7	0.0	17.2
Quintile 2	53.1 (6.7)	63.6	38.2	3.5 (6.5)	72.7	0.0	6.6
Quintile 3	80.7(8.2)	90.9	65.5	3.6 (4.2)	15.3	0.0	4.5
Quintile 4	112.2 (10.4)	136.4	91.6	3.4 (4.5)	29.1	0.0	3.0
Quintile 5	313.0 (312.8)	2181.8	141.8	5.5 (12.0)	72.7	0.0	1.8

() Standard deviations

Table 2 also indicates that overall, WTP increases as we move from a lower quintile to an upper quintile. WTP increased from 1881.8 FCFA (or USD 3.4) in quintile 1 (the poorest quintile) to 3959.5 FCFA (or USD 7.2) in quintile 5 (the richest quintile). However, in terms of proportion, ISW in the poorest quintiles are willing to allocate more important part of their income to the premium than the richest quintiles. For example, ISW in quintile 1 are willing to allocate on average, 15% of their income to the MHI while those in quintile 5 are willing to allocate only 2.5% of their income. Furthermore, women are willing to allocate a lower part of their income (3.7%) than men (5.1%). Similarly, ISW in urban areas are willing to allocate a lower part of their income (3.9%) than those residing in rural areas (5%).

Table 3 summarizes the distribution of WTP by sex and location. Overall, 92.7% of Togolese ISW are interested in health insurance. This result reveals that there is a strong demand for health insurance by ISW. However, the majority of workers (47.2%) are willing to pay a maximum premium of 1226 FCFA (USD 2.2) per month, which is at least equal to the monthly premium of a worker in the formal sector who earns the guaranteed minimum wage (SMIG) per month. Furthermore, 45.43% of ISW are willing to pay more than the premium of an ISW earning more than the minimum wage, and more than 16% are willing to pay more than thrice the premium of an employee earning the SMIG.

**Table 3:** Distribution of WTP by sex and location of residence

WTP (in US Dollars)	Urban (%)			Rural (%)			Total (%)		
	Men	Women	All	Men	Women	All	Men	Women	All
Not interested	6.9	6.5	6.6	9.1	7.9	8.5	8.0	6.9	7.3
Less than 2.2	36.7	47.4	43.8	45.4	58.8	52.4	40.9	51.3	47.2
2.2–3.8	21.9	20.0	20.7	22.2	20.4	21.2	22.0	20.2	20.9
3.8–7.6	8.7	9.0	8.9	10.0	4.6	7.2	9.3	7.5	8.2
7.6–19.1	23.7	16.1	18.6	13.0	8.1	10.5	18.5	13.4	15.4
More than 19.1	2.1	1.1	1.4	0.3	0.2	0.3	1.2	0.8	0.9

Table 4 shows the distribution of willingness to pay by income quintile, taking into account gender and location. As shown in the Table 4, outside the poorest quintile where the proportion of women not interested in MHI (14.1%) is higher than that of men (6.7%), women are more interested in MHI than men, regardless of the income quintile considered. Similarly, a higher proportion of women are willing to pay less than the premium of a formal sector worker earning the SMIG, regardless of the income quintile (61.5%; 61.0%; 43.3%; 51.2% and 48.8% respectively, from the poorest quintiles to the richest quintiles). These proportions are 51.4%; 41.8%; 39.7%; 38.8% and 36.8% respectively for men. As one moves towards the higher WTP brackets, the proportion of men tends to increase and that of women to decrease, meaning that the number of men willing to pay grows higher than that of women in almost all income quintiles. This finding confirms that women are generally more interested in MHI than men but that men are willing to pay higher premiums than women regardless of income levels.

**Table 4:** Distribution of WTP by income quintile (in %)

WTP in US dollars	Not interested		Less than 2.2		2.2–3.8		3.8–7.6		7.6–19.1		More than 19.1	
Income quintile												
	Men	Women	Men	Women	Men	Women	Men	Women	Men	Women	Men	Women
Very poor	6.7	14.1	51.4	61.5	24.8	15.6	1.9	3.0	15.2	5.9	0.0	0.0
Poor	8.2	5.2	41.8	61.0	23.3	19.9	8.2	5.6	17.8	8.2	0.7	0.0
Middle	6.9	9.0	39.7	43.3	23.7	24.2	9.9	11.2	18.3	12.4	1.5	0.0
Rich	8.2	5.3	38.8	51.2	17.0	21.8	15.0	7.1	20.4	14.1	0.7	0.6
Very rich	7.0	2.4	36. 8	48.8	23.2	16.1	9.2	5.4	21.1	23.8	2.7	3.6
Location												
	Urban	Rural	Urban	Rural	Urban	Rural	Urban	Rural	Urban	Rural	Urban	Rural
Very poor	13.6	4.8	31.8	56.6	40.9	20.5	0.0	2.4	13.6	15.7	0.0	0.0
Poor	7.0	9.3	42.2	41.3	21.1	25.3	7.0	9.3	21.1	14.7	1.4	0.0
Middle	6.3	7.3	36.5	42.6	23.8	23.5	7.9	11.8	22.2	14.7	3.2	0.0
Rich	6.2	10.6	35.8	42.4	18.5	15.1	12.3	18.2	27.2	12.1	0.0	1.5
Very rich	6.1	9.4	33.3	45.3	21.2	28.3	9.1	9.4	26.5	7.5	3.8	0.0

The analysis of WTP distribution by educational level (Table 5) shows that 90% of Togolese ISW with no level of education are interested in health insurance, compared to 94% of those with primary level education; and 93.8% of those with university level education.

**Table 5:** Willingness to pay according to the level of education of the household head

WTP in US Dollars	Educational level			
	None (%)	Primary (%)	Secondary (%)	University (%)
Not interested	10.0	6.0	5.8	6.2
Less than 2.2	55.6	45.4	40.9	37.5
2.2–3.8	16.6	24.6	22.6	18.7
3.8–7.6	5.9	10.4	9.1	4.2
7.6–19.1	11.3	12.0	20.7	33.3
More than 19.1	0.6	1.5	0.9	0.0
Average WTP (in US dollars)	1.5	1.8	2.0	2.2

### Determinants of willingness to pay

Table 6 summarizes the results of the Tobit model. Column (1) shows the results of the whole sample; then an analysis by gender of the head of the household is presented in columns (2) and (3); finally, columns (4) and (5) summaries the results by location of residence. At a 5% level, Table 6 (column (1)) reveals that the sex of the household head, the household size, education, income and square income are the main determinants of the ISWs’ willingness to pay for health insurance. The results indicate a negative correlation between sex (female) and WTP. We also found that when the household size is larger, the household head is willing to pay a higher premium. Otherwise, variables such as age, location, marital status of the head of household, number of children in the household, number of persons over 60 in the household, and distance to the nearest health facility have no significant effect on WTP.

**Table 6:** Result of the Tobit model

	(1)	(2)	(3)	(4)	(5)
Variables	All	Male	Female	Urban	Rural
Willingness to pay for MHI (WTP)					
Age	−14.1	−48.7	21.3	175.6**	−88.8
	(53.5)	(112.2)	(47.8)	(88.8)	(71.4)
Age2	−0.1	0.2	−0.3	−2.7**	0.9
	(0.6)	(1.3)	(0.5)	(1.1)	(0.8)
Rural area	− 227.2	− 205.0	−46.6		
	(282.2)	(528.2)	(273.2)		
Education Primary	55.7	136.6	121.7	430.9	− 444.6
	(298.6)	(610.2)	(271.2)	(402.5)	(445.5)
Secondary	423.1	821.0	77.1	596.1	344.6
	(308.4)	(596.7)	(291.4)	(393.9)	(505.2)
University	1,9***	2,4**	531.8	2,4***	−5,1
	(709.0)	(1,1)	(1,0)	(751.6)	(3,6)
Income	0.02***	0.03***	0.01***	0.02***	0.01**
	(0.0)	(0.0)	(0.0)	(0.0)	(0.0)
Income 2	−1.8e-08***	−4.0e-08***	−1.3e-08***	−2.2e-08***	− 1.5e-08*
	(3.2e-09)	(9.5e-09)	(2.7e-09)	(3.6e-09)	(8.1e-09)
Distance to health center: 100 m to less than 1 km	47.5	− 679.9	584.2*	233.9	− 649.1
	(332.7)	(636.4)	(314.7)	(390.6)	(693.7)
1 km to 3 km	−215.5	− 820.2	293.7	−43.4	− 278.7
	(323.3)	(604.8)	(310.0)	(430.1)	(492.1)
3 km to 5 km	− 632.9	− 1280	32.65	− 1071	− 513.7
	(611.6)	(1042)	(650.0)	(951.4)	(804.8)
More than 5 km	77.5	− 335.8	299.6	−826.1	−195.3
	(642.5)	(1207)	(608.8)	(4292)	(712.8)
Household size	261.5***	471.1**	154.9*	183.3	419.8***
	(97.9)	(193.7)	(91.6)	(124.6)	(157.5)
Number of old persons	−47.8	−323.9	−61.7	4018***	− 1261*
	(643.4)	(1429)	(551.2)	(1350)	(725.4)
Number of children	− 264.1	− 586.8	−82.9	−71.8	− 570.7*
	(231.5)	(462.8)	(211.9)	(324.0)	(327.8)
Marital status: Married	− 155.3	− 151.7	−264.8	− 249.5	− 21.9
	(331.9)	(621.8)	(322.6)	(383.6)	(664.2)
Divorced or separated	13.8	1844	− 681.0	519.5	− 666.8
	(602.2)	(1497)	(509.2)	(771.6)	(993.2)
Widow/widower	−68.1	1267	−643.6	257.4	−446.9
	(514.1)	(1447)	(440.1)	(666.7)	(873.8)
Gender: female	−805.2***			− 1022***	− 458.3
	(245.1)			(308.9)	(401.2)
Constant	1953*	2018	477.5	− 1699	3349**
	(1128)	(2213)	(1046)	(1711)	(1586)
Sigma					
	4281***	5414***	2979***	4240***	4214***
	(79.0)	(149.1)	(74.1)	(99.9)	(125.1)
Observations	1605	723	882	976	629

() Standard errors; *** *p* < 0.01, ** *p* < 0.05, * *p* < 0.1

The comparison of results by gender indicates that for female-headed households, income is the only variable that significantly explains WTP; household size is significant only at a 10% threshold. On the other hand, for male-headed households, apart from income, education and household size have a significant and positive effect on WTP. There are also some differences depending on location. In urban areas, the sex of the household head has a negative and significant effect on WTP, and no significant effect in rural areas. Similarly, in urban areas, income, age, number of people over 60 years of age, and education have a positive and significant effect on WTP. On the other hand, only income and household size have a significant effect on WTP in rural areas.

## Discussion

Our results show that men are willing to pay more than women and corroborate those of Dong et al. [28] who showed that in Burkina Faso (country bordering Togo), men were willing to pay a high amount for access to community insurance compared to women. Atake and Agbodji [12], Onwujekwe et al. [18] as well as Bärnighausen et al. [15] also arrived at the same conclusion. A possible explanation for this might be that women are poorer and more vulnerable than men in the informal sector. Indeed, Agbodji et al. [29], Djahini-Afawoubo [30], and Noglo [31] have shown that in Togo, women are more vulnerable and more affected by multidimensional poverty than men. Our results show that women in Togo have a low level of education, fewer job opportunities, less income, etc. compared to men. All these factors could explain the difference in willingness to pay between men and women. It is therefore important to take gender into account in extending mandatory health insurance, in order to ensure that in poor households, women’s lack of access to resources and inequitable decision-making power does not hinder their participation in MHI. Our findings suggest that, in extending the MHI project to the informal sector workers, it is also necessary to consider the possibility of providing highly subsidized or free health insurance for women, as a way of limiting the impoverishment of female-headed households.

Another finding of our study is that income, square income, education and household size are the main determinants of ISWs’ willingness to pay for health insurance. Income has a positive effect on WTP. This result corroborates our expectations. The demand theory predicts a positive elasticity income for most goods (except inferior goods). Previous studies have also found a similar result [15, 21, 32]. The sign of square income being negative suggests that the relation between income and WTP is not linear. For low income levels, WTP increases as income increases. But once a certain threshold is reached, when income increases, the household heads reduce the maximum premium amount they are willing to pay for health insurance. It is therefore likely that for incomes below the threshold, the household head considers that his household is more vulnerable to health care expenditures. In this regard, health insurance is valued and the household head agrees to pay a higher amount. On the other hand, when the income is above the threshold, the household head can cope more easily with important health care expenditures without encountering financial difficulties. He then considers that his household is less vulnerable to health care expenditures and becomes more unwilling to pay. In other words, he substitutes the consumption of other goods such as foodstuff for the consumption of health insurance.

One of the most interesting results is that heads of households with a higher education are willing to pay a higher premium compared to those with other levels of education. We found that primary and middle school education have no significant effect on WTP. Bärnighausen et al. [15] also found that an educational level above middle school positively and significantly influences WTP. From the Togolese context, we can deduce the existence of an educational disparity in the WTP for health insurance. Hence, the more educated people are better informed or better able to process the available information on accessibility to health insurance and its consequences in terms of financial protection. There is therefore a need to adopt a communications strategy to mitigate the effect of education on health insurance.

As Table 6 shows, the larger the household size, the more willing a household head is to pay a higher premium amount. These results contradict those of De Allegri et al. [32] who found that household size was an obstacle to community health insurance coverage in Burkina Faso, citing the financial burden that health insurance would create for all household members. The same is true of the conclusions reached by Jehu-Appiah et al. [33] to the effect that large households in Ghana were less likely to be enrolled in a health insurance scheme. The positive effect of household size on WTP in Togo can be explained by the vulnerability of informal employment. Most people working in the informal sector are also the most vulnerable [34]. Thus, given the irregular nature of their income, large households in the informal sector would be the most vulnerable to health expenditures [12, 35]. Access to health insurance would reduce catastrophic health expenditures in these types of households, improve health status and increase the productivity of their members [34].

Our results also reveal that over 92% of ISW would like to have access to health insurance, like those in the formal sector do. The advantages of health insurance would explain this high rate of ISW willingness’ to be insured [1, 8, 9]. Access to universal health insurance would enable all household members in the informal sector to access the health services they need without experiencing financial difficulties. Access to health services would also enable ISW, which accounts for about 30% of GDP, to be more productive and contribute more actively to family and community life. Our results suggest that one of the best ways to improve the well-being of the population and growth in Togo would be to extend mandatory health insurance to the informal sector. This proposal is supported by the fact that the lowest WTP indicated by informal sector workers is close to the premium of a formal sector worker earning the SMIG per month. These results suggest that health insurance for ISW is possible provided the State subsidizes the insurance of the formal and informal sector workers equally.

An important issue for policy makers is to know how much the state would need to subsidize the extension of MHI to ISW. To answer this question, we need information on income and the total number of informal sector households in the total population.

According to the National Institute of Statistics and Economic and Demographic Studies (INSEED), the total population of Togo in 2018 is estimated at 7,352,000 inhabitants or about 1,564,255 households. Knowing that the ISW represent 90.4% of the Togolese population, the ISW population can be estimated at 1,414,087 households.

ISW’s average income has been updated using the Central Bank of West African States (BCEAO) price index. Since the average income can be sensitive to extreme values, the ISW are grouped into income quintile to minimize this effect. Table 7 presents the updated average income and average WTP per quintile as well as the share of each quintile in the total ISW households. According to Table 7, ISW in the first two quintiles (41.8%) earn a monthly income lower than the SMIG. As a result they are not eligible for MHI. A strategy that fails to include them in the MHI would be inappropriate, for universal coverage purpose. Thereof, the state should include them in the MHI and entirely subsidize their premium.

**Table 7:** Distribution of WTP and income by quintile

	Share in the population (%)	Actualized Average Income (USD)			Actualized Average WTP (USD)		
		Men	Women	All	Men	Women	All
All households	100.0	131.8	120.4	126.0	6.7	4.4	5.4
quintile 1	20.2	27.3	25.7	26.5	5.0	2.5	4.0
quintile 2	21.6	63.3	62.5	62.9	4.5	3.6	4.2
quintile 3	19.9	95.9	93.9	95.0	5.0	4.4	5.0
quintile 4	19.2	131.6	131.4	131.5	4.8	4.6	5.1
quintile 5	19.1	316.9	345.3	329.2	8.4	6.8	8.4

Assuming that the state would subsidize the entire premium of ISW in the first two quintiles and 50% of the premium of the rest, the monthly amount that the state would need to subsidize can be calculated using the following equation:

5 $$ S=\sum \limits_{i=1}^5{Q}_i\times {Income}_i\times {\lambda}_i $$

Where *S* is the monthly subsidy,*Income*_*i*_, the updated average income of quintile *i, λ*_*i*_, the share of the state contribution paid to quintile *i*, and *Q*_*i*_, the total number of informal sector households in the quintile *i*. The annual subsidy can be then estimated by multiplying the monthly subsidy by 12. Based on eq. (5), the annual subsidy is estimated at 53,826,018,966 FCFA or 4.1% of the state current general budget and 96% of the health sector budget. This implies that the State should increase its total allocation towards health sector by 96% in order to be able to extend MHI to ISW. The budget allocated to the health sector currently represents only 4.2% of the State’s overall budget. Even if the state increases the health sector budget by 96%, it will not be able to respect the Abuja recommendation that requires each African State to allocate at least 15% of their budget to health sector. Summarizing, the MHI can be extended to ISW provided the state subsidizes the MHI by 4.1% of its current budget.

However, it’s important to emphasize the difficulty of Government to have reliable information on ISW income. Given that the state should fully cover the contribution of ISW with a monthly income below the SMIG, this can lead to free riding problem since some ISW would be encouraged to declare income below the SMIG in order to be entirely covered by the state. This involves designing differentiated health service packages according to the amount of each ISW’s premium, so that the higher the premium, the greater the number of health services included in the package. Such a measure would provide an incentive for high-income ISW to contribute significantly and thus guarantee the sustainability of the system.

### Limitations of the study

This study has some limitations. The data used to measure the various indicators was declared by the respondents themselves and cannot be verified using other administrative sources. We furthermore used income as an indicator to understand households’ living standards. However, it may have been more appropriate to use household consumption expenditure given that it is more difficult to measure the income of the self-employed, particularly the ISW, and that the respondents were most often reluctant to disclose their income. On the other hand, due to lack of information on household consumption expenditure, we had to use income as an indicator of the standard of living. Lastly, WTP must not be considered as an insurance premium as it is only an indication of the respondents’ willingness to pay for a given benefits package.

## Conclusion

The main objective of this paper was to assess the willingness of ISW to pay for MHI, and to analyze the determinants of WTP. We were thus able to analyze how MHI can be extended to ISW. We found that the extension of MHI to ISW is possible in Togo. It is clear from our findings that about 92% of ISW wish to be enrolled in MHI, like those in the formal sector. On average, ISW are willing to pay 2569 FCFA (USD 4.7), which is higher than the premium of a formal sector worker who earns twice the SMIG. 47.2% of ISW are willing to pay an amount that is less than or equal to the premium of an ISW earning the SMIG, while 45.4% are willing to pay a higher amount. Over 16% are willing to pay over three times as much as an employee earning the SMIG. Men are willing to pay on average a higher premium than women. Similarly, urban residents are willing to pay on average a higher amount than rural residents. The determinants of WTP were identified as: sex of the household head, household size, education and income. A number of economic policy recommendations emerged from our study, namely.

It is possible to extend health insurance to ISW, since over 92% are in favor of it, and since an ISW earns on average 59,726 FCFA (USD 108.59), which is 1.7 times the amount required to be enrolled in the MHI. Given that one must earn a salary that is at least equal to the SMIG in order to be enrolled in the MHI and that, on average, ISW agree to pay an amount that is higher than the premium of a formal sector employee who earns twice the SMIG, the extension of MHI to informal sector workers would not jeopardize the viability of MHI. However, for the sake of equity, the State should subsidize formal and informal sector workers equally to avoid creating double standards within the health insurance scheme. Currently, an employee’s premium to MHI amounts to 7% of their gross salary. The employee contributes 3.5% of his salary and the Government pays the other half. In the case of ISW, the Government should therefore adopt the same subsidy policy by paying half the premium of each worker.

Our results shown that the annual subsidy is estimated at 53,826,018,966 FCFA or 4.1% of the State’s current general budget or 96% of the health sector budget. This implies that the State should increase the budget allocated to the health sector by 96% in order to be able to extend MHI to ISW. With regard to tax revenues, the estimated annual subsidy represents about 8.1%. In the context of poverty and scarcity of resources, Togolese Government should develop resource mobilization strategies to fund the extension of the MHI to the ISW. For this purpose, Government could study the possibility to increase taxes on products such as tobacco, alcohol, airline tickets, financial transactions, etc. Some Asian countries such as the Philippines and India have successfully funded their universal health insurance system with high taxes on tobacco and alcohol [36, 37]. Moreover, Government could plead for donors funding. It is important to emphasize that resource mobilization and management, as well as health services delivery, would be effective only in a context of improved governance. [38]

Furthermore, the design of differentiated health service packages appear very important because it would provide an incentive for high-income ISW to contribute significantly and thus guarantee the sustainability of the system.

There is also a need to adopt a communications strategy to mitigate the effect of low level education on health insurance. With this regard, communication on ISWs’ access to MHI and its advantages should be done in local language or mother tongue. Such a program could involve every ISW. All awareness raising tools and manuals should be translated into the local languages.

## Abbreviations

CBMS: Community-Based Monitoring System
CNSS: National Social Security Fund
CRT: Pension Fund of Togo
DFID: Department for International Development
GDP: Gross Domestic Product
IDRC: International Development Research Center
INAM: National Institute of Health Insurance
ISW: Informal Sector Workers
MHI: Mandatory Health Insurance
OLS: Ordinary Least Squares
PEP: Partnership for Economic Policy
SMIG: guaranteed minimum wage
UK Aid: United Kingdom Aid
WTP: Willingness to Pay
